# A Panel of Serum Biomarkers Differentiates IgA Nephropathy from Other Renal Diseases

**DOI:** 10.1371/journal.pone.0098081

**Published:** 2014-05-23

**Authors:** Hiroyuki Yanagawa, Hitoshi Suzuki, Yusuke Suzuki, Krzysztof Kiryluk, Ali G. Gharavi, Kiyoshi Matsuoka, Yuko Makita, Bruce A. Julian, Jan Novak, Yasuhiko Tomino

**Affiliations:** 1 Division of Nephrology, Department of Internal Medicine, Juntendo University Faculty of Medicine, Tokyo, Japan; 2 Division of Nephrology, Department of Medicine, Columbia University College of Physicians and Surgeons, New York, New York, United States of America; 3 Clinical Research Center, Juntendo University Faculty of Medicine, Tokyo, Japan; 4 Department of Medicine, University of Alabama at Birmingham, Birmingham, Alabama, United States of America; 5 Department of Microbiology, University of Alabama at Birmingham, Birmingham, Alabama, United States of America; South Texas Veterans Health Care System and University Health Science Center San Antonio, United States of America

## Abstract

**Background and Objectives:**

There is increasing evidence that galactose-deficient IgA1 (Gd-IgA1) and Gd-IgA1-containing immune complexes are important for the pathogenesis of IgA nephropathy (IgAN). In the present study, we assessed a novel noninvasive multi-biomarker approach in the diagnostic test for IgAN.

**Materials and Methods:**

We compared serum levels of IgA, IgG, Gd-IgA1, Gd-IgA1-specific IgG and Gd-IgA1-specific IgA in 135 IgAN patients, 79 patients with non-IgAN chronic kidney disease (CKD) controls and 106 healthy controls. Serum was collected at the time of kidney biopsy from all IgAN and CKD patients.

**Results:**

Each serum marker was significantly elevated in IgAN patients compared to CKD (P<0.001) and healthy controls (P<0.001). While 41% of IgAN patients had elevated serum Gd-IgA1 levels, 91% of these patients exhibited Gd-IgA1-specific IgG levels above the 90th percentile for healthy controls (sensitivity 89%, specificity 92%). Although up to 25% of CKD controls, particularly those with immune-mediated glomerular diseases including lupus nephritis, also had elevated serum levels of Gd-IgA1-specific IgG, most IgAN patients had elevated levels of Gd-IgA1-specific antibody of both isotypes. Serum levels of Gd-IgA1-specific IgG were associated with renal histological grading. Furthermore, there was a trend toward higher serum levels of Gd-IgA1-specific IgG in IgAN patients with at least moderate proteinuria (≥1.0 g/g), compared to patients with less proteinuria.

**Conclusions:**

Serum levels of Gd-IgA1-specific antibodies are elevated in most IgAN patients, and their assessment, together with serum levels of Gd-IgA1, improves the specificity of the assays. Our observations suggest that a panel of serum biomarkers may be helpful in differentiating IgAN from other glomerular diseases.

## Introduction

IgA nephropathy (IgAN) is the most common type of primary glomerulonephritis worldwide [1], [2]. IgAN has a significant morbidity, culminating in end-stage kidney disease in about 40% of patients within 20 years of diagnosis [3]. Renal biopsy is required for the diagnosis of IgAN. Typical histological features include granular mesangial deposits of IgA, usually accompanied by C3, a variable presence of IgG and/or IgM, and diverse degrees of mesangial cellular proliferation and expansion of the extracellular matrix [4].

Several recent studies suggest that aberrant *O*-glycosylation of circulatory IgA1 is vital in the pathogenesis of IgAN. The *O*-linked glycans in the hinge region of IgA1 are generally composed of *N*-acetylgalactosamine (GalNAc) and galactose; sialic acid may be attached to either or both sugars. IgA1-producing cells secrete a mixture of IgA1 *O*-glycofoms. Studies in different populations have shown that IgAN patients have significantly higher levels of circulating IgA1 with galactose-deficient, *O*-linked, hinge-region glycans [5]–[9]. Depending on the population studied, 50–75% of IgAN patients have levels above the 90^th^ percentile for healthy controls. In addition, IgA1 eluted from renal tissues of IgAN patients also exhibits a galactose deficiency in the *O*-linked glycans in the hinge-region [10], [11].

The serum level of IgA1-containing circulating immune complexes is elevated in patients with IgAN [12]–[14]. These complexes contain galactose-deficient IgA1 (Gd-IgA1) bound by IgG and/or IgA antibodies [14], [15]. Recently, we have shown that the IgG auto-antibodies that recognize glycan-containing epitopes on Gd-IgA1 exhibit unique features in the complementarity-determining region 3 of the variable region of their heavy chains [16]. Furthermore, the serum levels of IgG autoantibodies specific for Gd-IgA1 correlated with disease severity, as assessed by magnitude of proteinuria. However, the serum levels of Gd-IgA1-containing circulating immune complexes may differ widely among IgAN patients [15]. Furthermore, some IgAN patients do not show glomerular deposition of IgG, but rather only IgA. Therefore, it is difficult to explain the pathogenesis of IgAN by an elevated serum level of glycan-specific antibodies of only the IgG isotype. These latter features may be explained by our observation that some patients with IgAN have complexes generated by glycan-specific antibodies of the IgA1 isotype [15]. Whereas the serum levels of IgA, Gd-IgA1 and glycan-specific IgG are higher in patients with IgAN compared to healthy controls, the levels of these parameters have not been systematically studied in patients with other forms renal disease with clinical features similar to those of IgAN. We therefore examined the prevalence of elevated serum levels of IgA, Gd-IgA1 and glycan-specific IgG and IgA in IgAN patients and a large cohort of CKD controls to assess the utility of these biomarkers for the non-invasive diagnosis of IgAN. Our data revealed that this panel of biomarkers is helpful in differentiating patients with IgAN from patients with other glomerular diseases.

## Materials and Methods

### Ethics Statement

This study was performed according to the Declaration of Helsinki and approved by the Ethics Review Committee of Juntendo University Faculty of Medicine. All study participants provided written informed consent.

### Patients and controls

A cross-sectional study was performed using serum samples collected at Juntendo University Hospital in Japan from 2006 to 2010 at the time of renal biopsy from 135 patients with IgAN and 79 patients with other renal diseases as shown in Table 1. We collected serum samples from 106 healthy volunteers who had never exhibited any abnormality by urinalysis in medical examinations from 2009 to 2011. All patients and healthy volunteers were Japanese, and the demographic and clinical features are summarized in Table 2. Histological prognostic stages of IgAN were assessed by the Clinical Guidelines for IgA Nephropathy in Japan (second version) [17], and are as follows: Group I: good prognosis, Group II: relatively good prognosis, Group III: relatively poor prognosis, and Group IV: poor prognosis.

**Table 1:** Renal diseases in chronic kidney disease (CKD) controls.

Renal disease	N
Diabetic nephropathy	33
Membranous nephropathy	9
Non-IgA proliferative nephropathy	9
Lupus nephritis	8
Minimal change nephrotic syndrome	6
Nephrosclerosis	4
Interstitial nephritis	2
Focal segmental glomerular sclerosis	2
ANCA-related nephritis	1
Scleroderma renal crisis	1
HCV-related nephritis	1
Fabry's disease	1
Acute post-streptococcal nephritis	1
Minor glomerular abnormality	1

**Table 2:** Characteristics of patients with IgAN and CKD controls.

	IgAN patients	CKD controls	Healthy controls
Numbers	135	79	106
Age (years)	34.6±11.9	50.9±19.4	32.6±5.2
Sex (male/female)	56/79	42/37	61/45
Serum creatinine (mg/dl)	0.93±0.53	2.26±2.52	ND
eGFR (ml/min/1.73 m^2^)	76.9±25.8	55.7±39.2	ND
Urine P/C (g/g)	0.65±0.84	3.01±3.19	ND
Hematuria	4.08±2.28	1.60±2.23	ND

Values are mean ±SD.

eGFR, estimated glomerular filtration rate; P/C, protein/creatinine ratio; ND, not determined.

Hematuria: Assessed by assigning scores according to number of red blood cells per high-power field (RBC/HPF).

≤5 RBC/HPF  = 0, 6–10 RBC/HPF  = 1, 11–15 RBC/HPF  = 2, 16–20 RBC/HPF  = 3, 21–25 RBC/HPF  = 4, 26–30 RBC/HPF  = 5, >30 RBC/HPF  = 6.

### Subgroups of disease controls

As the urinary protein/creatinine ratio in disease controls was higher than that in IgAN patients, we divided CKD controls into two subgroups: one with urinary protein/creatinine ratio ≥2.5 g/g (high-proteinuria) and the second with urinary protein/creatinine ratio <2.5 g/g (low-proteinuria). Then, we compared the levels of each biomarker between the two subgroups.

### Measurement of serum biomarkers

#### Serum level of Gd-IgA1

The serum level of Gd-IgA1 was measured by lectin ELISA using GalNAc-specific lectin from *Helix aspersa* (HAA; Sigma, St. Louis, MO) as previously reported [9], [18], [19]. Diluted sera were added 100 ng per well of serum IgA. The captured IgA was treated with 10 mU/ml neuraminidase (Roche Diagnostic Corp. Indianapolis, IN) to remove terminal sialic acid residues [9], [19]. The desialylated IgA1 was then reacted with biotin-labeled HAA and subsequently developed; absorbance was measured at 490 nm. The HAA reactivity of IgA1 in each sample was then calculated as OD units/100 ng of serum IgA. Naturally galactose-deficient IgA1 (Ale) myeloma protein [9] treated with neuraminidase and was used as the standard. Serum level of total Gd-IgA1 was expressed in relative Units, calculated by multiplying the normalized HAA reactivity by the amount of IgA in the serum sample (mg/ml).

#### Serum level of Gd-IgA1-specific IgG

ELISA plates were coated with the Fab fragment of Gd-IgA1 myeloma protein (Ste) generated with an IgA-specific protease from *Haemophilus influenzae* HK50 [15]. The amount of total IgG used for the analyses was normalized in all samples and added to each well. Captured IgG was detected with a biotin-labeled F(ab')_2_ fragment of goat IgG anti-human IgG antibody (BioSource; Invitrogen, San Diego, CA). Avidin–horseradish peroxidase conjugate (ExtrAvidin; Sigma-Aldrich) was then added, and the reaction was developed [16]. Serum levels of Gd-IgA1-specific IgG were expressed in Units (1 unit as OD 1.0 measured at 490 nm).

#### Serum level of Gd-IgA1-specific IgA

ELISA plates were coated with Fab fragment of Gd-IgA1 (Ste) described above [15]. Captured antibodies were detected by incubation with mouse monoclonal antibody to human IgA (Fc-specific) (Applied Biological Materials Inc., Richmond, BC) and detected by Peroxidase-conjugated AffiniPure Goat Anti-Mouse IgG (H+L) (Jackson Immuno Research, West Grove, PA). Serum levels of Gd-IgA1-specific IgA were expressed in Units (1 unit as OD 1.0 measured at 490 nm).

### Statistical analysis

Data are expressed as means ± SD. Comparison of groups was performed using univariate ANOVA, and *post hoc* Bonferroni correction was used for multiple comparisons. Correlation between two groups was performed by regression analysis. *P*<0.05 was considered significant. These statistical analyses were performed using the Prism software (GraphPad Software Inc., La Jolla, CA). The discriminatory ability of the biomarkers and clinical data combination was estimated with the area under the receiver operating characteristic curve (AUC-ROC). We additionally used Akaike's Information Criterion (AIC) to select the best predictive model [20]. The sensitivity, specificity, and positive and negative predictive values were calculated based on the ROC curves with the cut-off selected at the 90^th^ percentile biomarker value for healthy controls. We interpreted the model with the lowest AIC as the most useful in differentiating cases from controls. These analyses were performed with IBM SPSS Statistics release 19.0.

## Results

### Clinical profiles, serum IgA, IgG and Gd-IgA1

The present study was comprised of 135 IgAN patients, 79 CKD controls and 106 healthy controls (Table 2). Age, serum creatinine concentration, and the urinary protein/creatinine ratio were higher for the CKD controls than for IgAN patients.

The serum IgA concentration was significantly higher for the IgAN patients (3.60±1.47 mg/ml) compared to those of the CKD controls (2.71 ±1.04 mg/ml, P<0.001) and healthy controls (2.17±0.75 mg/ml, P<0.001). Half of the patients with IgAN had a serum IgA concentration higher than the 90^th^ percentile for healthy controls (3.06 mg/ml), consistent with a prior publication [21]. There was no significant difference in serum IgG concentration between the different groups.

**Figure 1:**
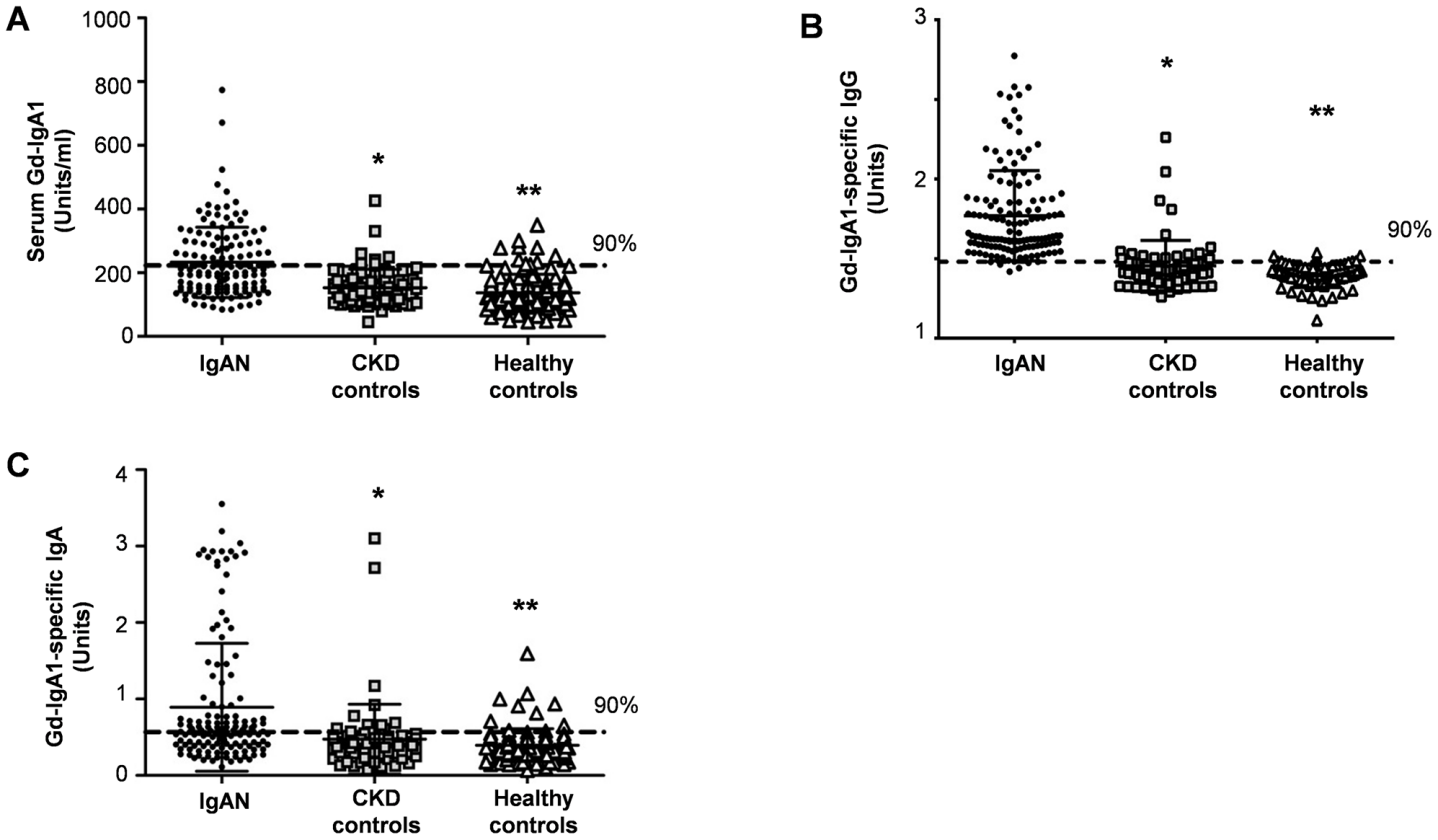
Distribution of serum levels of (A) Gd-IgA1, (B) Gd-IgA1-specific IgG and (C) Gd-IgA1-specific IgA in patients with IgAN (n = 135), CKD controls (n = 79) and healthy controls (n = 106). Each biomarker was measured by capture ELISA. The serum levels of Gd-IgA, Gd-IgA1-specific IgG and Gd-IgA1-specific IgA were higher in IgAN patients compared with those of the CKD controls (*P<0.001) and healthy controls (**P<0.0001).

Also consistent with prior reports, the serum level of Gd-IgA1 for the IgAN patients was significantly higher compared to that for the CKD controls (P<0.001) and healthy controls (P<0.001) (Figure 1A). Fifty-six of 135 IgAN patients (41%) but only eight nineof 79 (11%) CKD controls had a serum Gd-IgA1 level higher than the 90^th^ percentile for healthy controls (P = 5.5×10^-7^ for differences in distribution in IgAN patients *vs.* CKD controls).

### Serum levels of IgG and IgA antibodies against Gd-IgA1

IgAN patients had significantly higher levels of serum Gd-IgA1-specific IgG compared with those of the CKD controls (P<0.001) and healthy controls (P<0.001) (Figure 1B). Most IgAN patients (123/135, 91%) had a serum level of Gd-IgA1-specific IgG higher than the 90^th^ percentile for healthy controls (1.48 Units). The differences in the distribution of Gd-IgA1-specific IgG in IgAN patients *vs.* disease controls were highly significant (P = 3×10^−24^).

Serum levels of Gd-IgA1-specific IgA were elevated in IgAN patients compared with CKD controls (P<0.001) and healthy controls (P<0.001) (0.890±0.840 Units for IgAN patients, 0.482 ±0.483 Units for CKD controls and 0.419±0.289 Units for healthy controls; Figure 1C). The serum level of Gd-IgA1-specific IgA was higher than the 90^th^ percentile for healthy controls (0.611 Units) for 43% of IgAN patients and 14% of CKD controls.

While the serum levels of Gd-IgA1-specific IgG or IgA were significantly higher in IgAN patients, about 25% of CKD controls also had a serum level of Gd-IgA1-specific IgG higher than the 90^th^ percentile for healthy controls. Many of IgAN patients (56/135, 41%) had elevated levels of both Gd-IgA1-specific IgG and IgA. In contrast, elevated levels of both IgG and IgA antibody against Gd-IgA1 were seen in only 5 of 79 CKD controls who had an immune- mediated glomerulonephritis, such as lupus nephritis or membranous nephropathy (Table 3). Twenty-three of 38 CKD controls with an immune-mediated kidney disease had an elevated serum level of Gd-IgA1-specific IgG or IgA. In contrast, only 8 of 41 CKD controls with non-immune-mediated renal disease had an elevated serum level of Gd-IgA1-specific IgG or IgA (p = 6×10^−4^ for differences in distribution between the two groups).

**Table 3:** Serum levels of Gd-IgA1-specific antibodies in patients with IgAN, CKD controls and healthy controls.

	Gd-IgA1-specific IgG (Units)	Gd-IgA1-specific IgA (Units)
IgAN (N = 135)	1.77±0.28	0.89±0.84
Lupus nephritis WHO class III (N = 2)	1.57±0.09	0.47±0.26
Lupus nephritis WHO class IV (N = 4)	1.62±0.19	0.47±0.20
Lupus nephritis WHO class V (N = 2)	1.91±0.49	0.50±0.04
Membranous nephropathy (N = 9)	1.52±0.19	0.48±0.20
Non-IgA proliferative glomerulonephritis (N = 9)	1.43±0.10	0.36±0.15
Diabetic nephropathy (N = 33)	1.43±0.08	0.35±0.13
Nephrosclerosis (N = 4)	1.40±0.07	0.44±0.33
Minimal-change nephrotic syndrome (N = 6)	1.52±0.27	0.66±0.28
Interstitial nephritis (N = 2)	1.33±0.02	0.58±0.01
Other immune mediated renal diseases (N = 6)	1.47±0.07	0.71±0.75
Other non-immune mediated renal diseases (N = 2)	1.39±0.09	0.59±0.10
Healthy controls (N = 106)	1.39±0.07	0.42±0.29

WHO, World Health Organization.

### Relationship between Gd-IgA1 and Gd-IgA1-specific IgG

**Figure 2:**
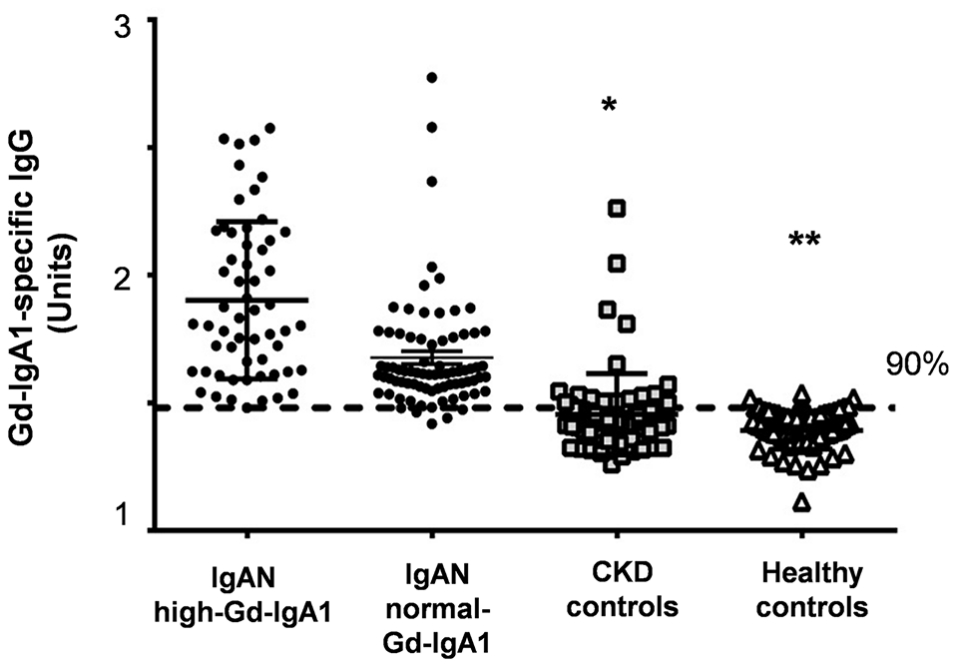
Serum levels of Gd-IgA1-specific IgG in IgAN patients with high-Gd-IgA1 or normal-Gd-IgA1 in comparison to CKD and healthy controls. We divided IgAN patients into two subgroups: patients with serum levels of Gd-IgA1 ≥ the 90^th^ percentile for healthy controls (high-Gd-IgA1 group; n = 56) and patients with levels Gd-IgA1 < the 90^th^ percentile for healthy controls (normal-Gd-IgA1 group; n = 79). Although serum levels of Gd-IgA1-specific IgG were significantly higher in IgAN patients with high Gd-IgA1 levels (*vs.* CKD controls; *P<0.0001, *vs.* healthy controls; **P<0.0001), IgAN patients with normal Gd-IgA1 levels also had elevated Gd-IgA1-specific IgG (*vs.* CKD controls; *P<0.0001, *vs.* healthy controls; **P<0.0001).

The serum levels of Gd-IgA1-specific IgG were significantly higher in IgAN patients with elevated Gd-IgA1 levels (P<0.001). However, about 91% of IgAN patients with a normal serum Gd-IgA1 level also had an elevated level of Gd-IgA1-specific IgG, suggesting that auto-antibody production may occur independently of serum Gd-IgA1 levels (Figure 2).

### Comparison of biomarker levels with clinical and pathological findings

We assessed each biomarker for possible correlation with histological findings and the clinical manifestations at the time of renal biopsy. Proteinuria was inversely correlated with serum immunoglobulin levels (IgA, and Gd-IgA1-specific IgG and IgA autoantibodies) in CKD controls (r = −0.17 to −0.28). This association was most strongly driven by the CKD controls, who had significantly more proteinuria than did the IgAN patients. To assess whether the serum levels of these immunogloubulins differed based on the magnitude of proteinuria, we divided the CKD disease controls into high-proteinuria and low-proteinuria subgroups, and compared the levels of each biomarker between the two subgroups. There was no significant difference in serum levels of IgA, Gd-IgA1, Gd-IgA1-specific IgG, and Gd-IgA1-specific IgA between the two subgroups (Table S1).

**Figure 3:**
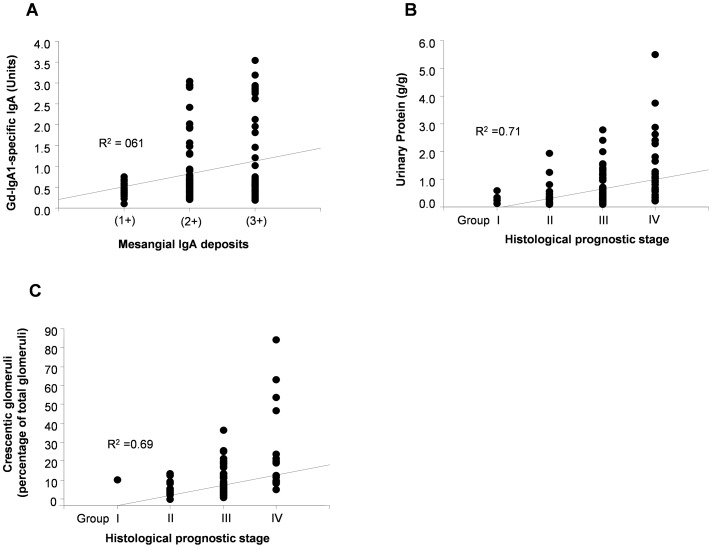
Correlation between biomarkers, histological findings and clinical findings. The strength of correlation between biomarkers, histological findings and clinical findings was measured by the Spearman's correlation coefficient. The serum level of Gd-IgA1-specific IgA correlated with the amount of mesangial IgA deposits (A). Histological prognostic stage (Clinical Guidelines for IgA Nephropathy in Japan, second version) [17] correlated with the urinary protein/creatinine ratio (B), and percentage of glomeruli with a crescent (C).

The serum level of Gd-IgA1-specific IgA correlated with degree of mesangial IgA deposits in patients with IgAN (R^2^ = 0.61) (Figure 3A). In addition, the histological prognostic stage according to the clinical guidelines for IgAN in Japan [17] correlated with proteinuria (R^2^ = 0.71) (Figure 3B) and the percentage of glomeruli with crescents (R^2^ = 0.69) (Figure 3C). As we previously reported [16], there was no significant correlation between the serum levels of Gd-IgA1-specific IgG and the degree of mesangial IgG deposition. However, there was a trend for the serum levels of Gd-IgA1-specific IgG to be higher in patients with greater mesangial IgG deposition (2+) (1.937±0.464 Units, n = 9) than in patients with no mesangial IgG deposition (1.747±0.326 Units, n = 13). Furthermore, the mean serum level of Gd-IgA1-specific IgG in the histological prognostic group 4 (n = 35, 1.821±0.318 Units) was high, compared to that in the histological prognostic group 1 (n = 8, 1.663±0.107 Units).

### Utility of biomarkers for non-invasive diagnosis of IgAN

**Figure 4:**
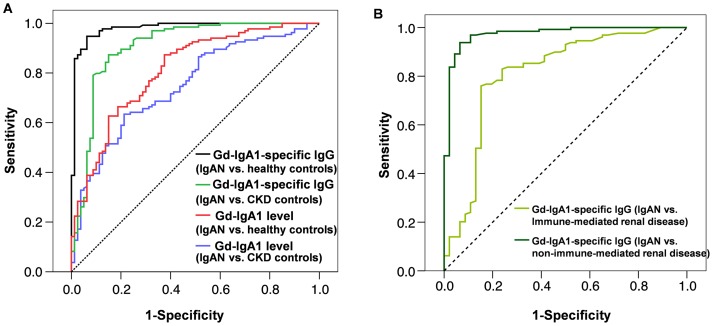
Receiver operating characteristic (ROC) curves. (A) Discrimination between IgAN versus healthy and CKD controls for serum Gd-IgA1 and Gd-IgA1-specific IgG levels; (B) Discrimination between IgAN versus CKD controls with immune-mediated renal disease and non-immune-mediated renal disease for serum Gd-IgA1-specific IgG levels.

We estimated the sensitivity and specificity of each biomarker for prediction of IgAN, using the cut-off value of the 90^th^ percentile for healthy controls. The serum level of Gd-IgA1-specific IgG performed best, providing a sensitivity of 89% and specificity of 92% for diagnosis of IgAN. The ROC curves showed excellent performance in comparison of IgAN vs. healthy controls (Figure 4, with corresponding statistics summarized in Tables 4 and 5). The areas under the ROC curve (C-statistics) for anti-Gd-IgA1 IgG were 0.965 (95%CI: 0.943–0.987) for discriminating IgAN cases *vs.* healthy controls, 0.973 (95%CI: 0.948–0.999) for non-immune-mediated CKD controls, and 0.813 (95%CI: 0.730–0.895) for immune-mediated CKD controls. In addition, the AIC for models testing Gd-IgA1-specific IgG were 57.4 for discriminating IgAN cases *vs.* healthy controls, 75.3 for non-immune-mediated CKD controls, and 145.2 for immune-mediated CKD controls.

**Table 4:** Statistics summarized data; Discrimination between IgAN versus healthy and CKD controls for serum Gd-IgA1 and Gd-IgA1-specific IgG levels.

	Area under the ROC curve (C-statistic)	Asymptotic 95% Confidence Interval for the C-statistic		Asymptotic Significance (P-value)	AIC***
		Lower Bound	Upper Bound		
Gd-IgA1-specific IgG (IgAN vs. healthy controls)	0.965	0.943	0.987	1.7E-35	57.4
Gd-IgA1-specific IgG (IgAN vs. CKD controls)	0.906	0.858	0.953	3.2E-23	188.5
Gd-IgA1-specific IgG (IgAN vs. CKD non-immune-mediated renal disease*)	0.973	0.948	0.999	1.7E-21	75.3
Gd-IgA1-specific IgG (IgAN vs. CKD immune-mediated renal disease**)	0.813	0.730	0.895	3.2E-10	145.2
Gd-IgA1 (IgAN vs. healthy controls)	0.800	0.745	0.855	9.9E-16	264.6
Gd-IgA1 (IgAN vs. CKD controls)	0.749	0.683	0.815	1.2E-09	246.3
Gd-IgA1-specific IgA (IgAN vs. healthy controls)	0.722	0.659	0.786	2.7E-09	295.6
Gd-IgA1-specific IgA (IgAN vs. CKD controls)	0.690	0.619	0.761	3.5E-06	266.1

* CKD non-immune-mediated renal disease includes diabetic nephropathy, nephrosclerosis, interstitial nephritis and Fabry's disease.

** CKD immune-mediated renal disease includes lupus nephritis, membranous nephropathy, minimal change disease, membranoproliferative glomerulonephritis, other types of non-IgAN glomerulonephritis.

***AIC: Akaike's Information Criterion.

**Table 5:** Statistics summarized data; Discrimination between IgAN versus immune-mediated CKD and non-immune-mediated CKD controls for serum Gd-IgA1-specific IgG levels.

	Sensitivity	Specificity	PPV	NPV
Gd-IgA1-specific IgG (IgAN vs. healthy controls)	89%	92%	92%	89%
Gd-IgA1-specific IgG (IgAN vs. all CKD controls)	89%	81%	82%	88%
Gd-IgA1-specific IgG (IgAN vs. non-immune-mediated CKD)	89%	96%	96%	90%
Gd-IgA1-specific IgG (IgAN vs. immune-mediated CKD)	85%	67%	72%	82%
Gd-IgA1 (IgAN vs. healthy controls)	41%	91%	82%	61%
Gd-IgA1-specific IgA (IgAN vs. healthy controls)	34%	92%	81%	58%

PPV, Positive Predictive Value; NPV, Negative Predictive Value.

These data suggest that although serum levels of Gd-IgA1-specific autoantibodies have excellent predictive properties to discriminate IgAN patients from healthy controls and patients with non-immune-mediated kidney disease, the specificity is lower for discriminating IgAN patients from patients with other types of immune-mediated kidney disease. Altogether, elevated Gd-IgA1-specific IgG provided positive predictive values of 92%, 96%, and 72%, and negative predictive values of 89%, 90%, and 82% for healthy controls and patients with non-immune kidney disease, and immune-mediated kidney disease, respectively.

## Discussion

IgAN is the most common type of primary glomerulonephritis worldwide and is often detected by urinary abnormalities, such as microscopic hematuria and low-grade proteinuria. The clinical features of IgAN vary between patients with IgAN. In biopsy specimens, glomerular deposits of IgA1 are sometimes accompanied by IgG and/or IgM. Although microscopic hematuria and low-grade proteinuria are frequent laboratory findings in patients with IgAN, some cases manifest a rapidly progressive clinical course with crescentic glomerulonephritis while others exhibit acute nephritic syndrome or nephrotic syndrome. The long-term prognosis is not benign; about 40% of IgAN patients progress to end-stage kidney disease within 20 years [3]. Thus, early diagnosis and treatment are necessary to prevent or slow disease progression. A noninvasive approach for the diagnosis of IgAN has been sought worldwide, especially in countries where the routine immunohistological processing of renal biopsy tissue is prohibitively expensive.

A pathway of multiple events has been proposed for the pathogenesis of IgAN [16], [22]. Immune complexes formed by the binding of Gd-IgA1 by Gd-IgA1-specific IgG or IgA in the circulation deposit in the mesangium and activate resident cells [12], [23]–[26]. Gd-IgA1-containing immune complexes from sera of patients with IgAN induce proliferation of human mesangial cells in culture whereas Gd-IgA1 alone does not [27], [28]. Activated mesangial cells produce inflammatory cytokines that cause glomerular injury [29], [30]. These reports suggest that Gd-IgA1-containing immune complexes play an essential role in the pathogenesis of IgAN. Therefore, we measured serum levels of the key biomarkers, IgA, Gd-IgA1, Gd-IgA1-specific IgG and Gd-IgA1-specific IgA to assess their utility in distinguishing patients with IgAN from patients with other forms of kidney disease and healthy controls.

The present study showed that serum levels of IgA, Gd-IgA1, Gd-IgA1-specific IgG and Gd-IgA1-specific IgA were elevated in patients with IgAN compared with those of healthy and CKD controls, suggesting that these parameters may be useful for the diagnosis of IgAN. However, several interesting limitations of these tests have emerged.

First, elevated serum levels of Gd-IgA1 have been found in healthy relatives of individuals with IgAN, suggesting that such levels alone are not sufficient to cause the disease [31]. Among patients with an established diagnosis of IgAN, the serum levels of Gd-IgA1 are variable [9]. Notably, longitudinal trajectories of serum Gd-IgA1 levels have not yet been established in prospective cohorts of IgAN cases, and some variability may also be expected when the diagnosis (the time of renal biopsy and serum sampling) occurs at different stages of the disease process.

Second, in this study, we frequently observed elevated levels of Gd-IgA1-specific autoantibodies in IgAN patients with normal serum Gd-IgA1 levels. One potential explanation of these data is that even when the circulating level of Gd-IgA1 is normal, there may be a sufficient number of galactose-deficient residues on these molecules to induce formation of pathogenic immune complexes in the setting of elevated Gd-IgA1-specific antibodies. Furthermore, because the *O*-glycosylation defects may occur preferentially at specific amino acid positions, [32] and the lectin-based assay is not able to discriminate which sites are involved, some pathogenic glycosylation defects may exist below the detection level of our lectin-based method.

We also found that serum levels of Gd-IgA1-specific IgA correlated with the intensity of mesangial IgA1 deposits, implying that Gd-IgA1-specific IgA plays a role in glomerular deposition of IgA1-containing immune complexes. Lastly, serum levels of Gd-IgA1-specific IgG associated with histological grading, and we detected a trend for higher serum levels of Gd-IgA1-specific IgG in IgAN patients with at least moderate proteinuria (≥1.0 g/g), when compared to patients with less proteinuria. These data suggest that Gd-IgA1-specific IgG may represent a marker of disease severity, but its prognostic utility will require validation in independent prospective cohorts.

We noted a substantial overlap in serum levels of individual biomarkers between patients with IgAN, CKD controls, and healthy controls. Consequently, no single biomarker was sufficiently specific for IgAN. The levels of Gd-IgA1-specific IgG and IgA were particularly elevated in patients with non-IgAN immune-mediated kidney disease, such as lupus nephritis in which increased propensity to auto-antibody production is well established. Notably, some of these autoantibodies may be polyreactive, *i.e.,* binding to several autoantigens [33], [34], thus complicating the assay for IgAN. Our data raise a question as to whether the anti-Gd-IgA1 autoantibodies in patients with lupus nephritis are detected as an artifact of polyreactivity or constitute pathogenic mediators of kidney injury in non-IgAN kidney disease. Additional studies are necessary to examine this aspect of non-IgAN glomerular diseases.

Another limitation of our study was that the CKD control group did not contain sufficient numbers of patients with glomerular disorders that are clinically difficult to differentiate from IgAN, such as Alport syndrome, thin basement membrane disease, post-infectious glomerulonephritis or membranoproliferative glomerulonephritis. Because of the relative rarity of these conditions, large multicenter studies will be required to validate our results in these settings.

## Conclusions

Serum levels of Gd-IgA1-specific antibodies are elevated in most IgAN patients, even in the setting of normal serum level of Gd-IgA1. Our study suggests that a panel of serum biomarkers may be helpful in differentiating IgAN from other glomerular diseases.

## Supporting Information

Table S1 **Serum levels of biomarkers in high-proteinuria and low-proteinuria CKD control subgroups.**
(PDF)

## References

[1] D'Amico G (1987) The commonest glomerulonephritis in the world: IgA nephropathy. Q J Med 64: 709–727. 3329736

[2] Julian BA, Waldo FB, Rifai A, Mestecky J (1988) IgA nephropathy, the most common glomerulonephritis worldwide. A neglected disease in the United States? Am J Med 84: 129–132. 10.1016/0002-9343(88)90019-8 3337116

[3] D'Amico G (2000) Natural history of idiopathic IgA nephropathy: role of clinical and histological prognostic factors. Am J Kidney Dis 36: 227–237. 10922300 10.1053/ajkd.2000.8966

[4] Russell MW, Mestecky J, Julian BA, Galla JH (1986) IgA-associated renal diseases: antibodies to environmental antigens in sera and deposition of immunoglobulins and antigens in glomeruli. J Clin Immunol 6: 74–86. 3514654 10.1007/BF00915367

[5] Mestecky J, Tomana M, Crowley-Nowick PA, Moldoveanu Z, Julian BA, et al (1993) Defective galactosylation and clearance of IgA1 molecules as a possible etiopathogenic factor in IgA nephropathy. Contrib Nephrol 104: 172–182. 8325028 10.1159/000422410

[6] Allen AC, Harper SJ, Feehally J (1995) Galactosylation of *N*- and *O*-linked carbohydrate moieties of IgA1 and IgG in IgA nephropathy. Clin Exp Immunol 100: 470–474. 7774058 10.1111/j.1365-2249.1995.tb03724.x PMC1534466

[7] Novak J, Julian BA, Tomana M, Mestecky J (2008) IgA glycosylation and IgA immune complexes in the pathogenesis of IgA nephropathy. Semin Nephrol 28: 78–87. 18222349 10.1016/j.semnephrol.2007.10.009 PMC2241661

[8] Hiki Y, Horii A, Iwase H, Tanaka A, Toda Y, et al (1995) *O*-linked oligosaccharide on IgA1 hinge region in IgA nephropathy. Fundamental study for precise structure and possible role. Contrib Nephrol 111: 73–84. 7758349

[9] Moldoveanu Z, Wyatt RJ, Lee JY, Tomana M, Julian BA, et al (2007) Patients with IgA nephropathy have increased serum galactose-deficient IgA1 levels. Kidney Int 71: 1148–1154. 17342176 10.1038/sj.ki.5002185

[10] Hiki Y, Odani H, Takahashi M, Yasuda Y, Nishimoto A, et al (2001) Mass spectrometry proves under-*O*-glycosylation of glomerular IgA1 in IgA nephropathy. Kidney Int 59: 1077–1085. 11231363 10.1046/j.1523-1755.2001.0590031077.x

[11] Allen AC, Bailey EM, Brenchley PE, Buck KS, Barratt J, et al (2001) Mesangial IgA1 in IgA nephropathy exhibits aberrant *O*-glycosylation: observations in three patients. Kidney Int 60: 969–973. 11532091 10.1046/j.1523-1755.2001.060003969.x

[12] Czerkinsky C, Koopman WJ, Jackson S, Collins JE, Crago SS, et al (1986) Circulating immune complexes and immunoglobulin A rheumatoid factor in patients with mesangial immunoglobulin A nephropathies. J Clin Invest 77: 1931–1938. 3711340 10.1172/JCI112522 PMC370554

[13] Coppo R, Basolo B, Piccoli G, Mazzucco G, Bulzomì MR, et al (1984) IgA1 and IgA2 immune complexes in primary IgA nephropathy and Henoch-Schönlein nephritis. Clin Exp Immunol 57: 583–590. 6467681 PMC1536277

[14] Tomana M, Matousovic K, Julian BA, Radl J, Konecny K, et al (1997) Galactose-deficient IgA1 in sera of IgA nephropathy patients is present in complexes with IgG. Kidney Int 52: 509–516. 9264010 10.1038/ki.1997.361

[15] Tomana M, Novak J, Julian BA, Matousovic K, Konecny K, et al (1999) Circulating immune complexes in IgA nephropathy consist of IgA1 with galactose-deficient hinge region and antiglycan antibodies. J Clin Invest 104: 73–81. 10393701 10.1172/JCI5535 PMC408399

[16] Suzuki H, Fan R, Zhang Z, Brown R, Hall S, et al (2009) Aberrantly glycosylated IgA1 in IgA nephropathy patients is recognized by IgG antibodies with restricted heterogeneity. J Clin Invest 119: 1668–1677. 19478457 10.1172/JCI38468 PMC2689118

[17] Tomino Y, Sakai H (2003) Special Study Group (IgA Nephropathy) on Progressive Glomerular Disease: Clinical guidelines for immunoglobulin A (IgA) nephropathy in Japan, second version. Clin Exp Nephrol 7: 93–97. 14586726 10.1007/s10157-003-0232-4

[18] Moore JS, Kulhavy R, Tomana M, Moldoveanu Z, Suzuki H, et al (2007) Reactivities of *N*-acetylgalactosamine-specific lectins with human IgA1 proteins. Mol Immunol 44: 2598–2604. 17275907 10.1016/j.molimm.2006.12.011 PMC2788496

[19] Suzuki H, Moldoveanu Z, Hall S, Brown R, Vu HL, et al (2008) IgA1-secreting cell lines from patients with IgA nephropathy produce aberrantly glycosylated IgA1. J Clin Invest 118: 629–639. 18172551 10.1172/JCI33189 PMC2157566

[20] Akaike H (1974) A new look at the statistical model identification. IEEE Trans Automat Contr 19: 716–723.

[21] van der Boog PJ, van Kooten C, van Seggelen A, Mallat M, Klar-Mohamad N, et al (2004) An increased polymeric IgA level is not a prognostic marker for progressive IgA nephropathy. Nephrol Dial Transplant 19: 2487–2493. 15252166 10.1093/ndt/gfh394

[22] Suzuki H, Kiryluk K, Novak J, Moldoveanu Z, Herr AB, et al (2011) The pathophysiology of IgA nephropathy. J Am Soc Nephrol 22: 1795–1803. 21949093 10.1681/ASN.2011050464 PMC3892742

[23] Coppo R, Amore A (2004) Aberrant glycosylation in IgA nephropathy (IgAN). Kidney Int 65: 1544–1547. 15086888 10.1111/j.1523-1755.2004.05407.x

[24] Barratt J, Feehally J (2005) IgA nephropathy. J Am Soc Nephrol 16: 2088–2097. 15930092 10.1681/ASN.2005020134

[25] Julian BA, Novak J (2004) IgA nephropathy: an update. Curr Opin Nephrol Hypertens 13: 171–179. 15202611 10.1097/00041552-200403000-00005

[26] Novak J, Julian BA, Tomana M, Mesteck J (2001) Progress in molecular and genetic studies of IgA nephropathy. J Clin Immunol 21: 310–327. 11720004 10.1023/a:1012284402054

[27] Novak J, Vu HL, Novak L, Julian BA, Mestecky J, et al (2002) Interactions of human mesangial cells with IgA and IgA-containing immune complexes. Kidney Int 62: 465–475. 12110007 10.1046/j.1523-1755.2002.00477.x

[28] Novak J, Tomana M, Matousovic K, Brown R, Hall S, et al (2005) IgA1-containing immune complexes in IgA nephropathy differentially affect proliferation of mesangial cells. Kidney Int 67: 504–513. 15673298 10.1111/j.1523-1755.2005.67107.x

[29] Lai KN, Leung JC, Chan LY, Saleem MA, Mathieson PW, et al (2008) Activation of podocytes by mesangial-derived TNF-alpha: glomerulo-podocytic communication in IgA nephropathy. Am J Physiol Renal Physiol 294: F945–955. 18256312 10.1152/ajprenal.00423.2007

[30] Lai KN, Leung JC, Chan LY, Saleem MA, Mathieson PW, et al (2009) Podocyte injury induced by mesangial-derived cytokines in IgA nephropathy. Nephrol Dial Transplant 24: 62–72. 18685143 10.1093/ndt/gfn441

[31] Gharavi AG, Moldoveanu Z, Wyatt RJ, Barker CV, Woodford SY, et al (2008) Aberrant IgA1 glycosylation is inherited in familial and sporadic IgA nephropathy. J Am Soc Nephrol 19: 1008–1014. 18272841 10.1681/ASN.2007091052 PMC2386728

[32] Takahashi K, Wall SB, Suzuki H, Smith AD4th, Hall S, et al (2010) Clustered O-glycans of IgA1: defining macro- and microheterogeneity by use of electron capture/transfer dissociation. Mol Cell Proteomics 9: 2545–2557. 20823119 10.1074/mcp.M110.001834 PMC2984237

[33] Zhang J, Jacobi AM, Wang T, Berlin R, Volpe BT, et al (2009) Polyreactive autoantibodies in systemic lupus erythematosus have pathogenic potential. J Autoimmun 33: 270–274. 19398190 10.1016/j.jaut.2009.03.011 PMC2783480

[34] Mietzner B, Tsuiji M, Scheid J, Velinzon K, Tiller T, et al (2008) Autoreactive IgG memory antibodies in patients with systemic lupus erythematosus arise from nonreactive and polyreactive precursors. Proc Natl Acad Sci USA 105: 9727–9732. 18621685 10.1073/pnas.0803644105 PMC2474524

