# Supplementary material for: A Panel of Serum Biomarkers Differentiates IgA Nephropathy from Other Renal Diseases
Source: PLoS One. 2014 May 23;9(5):e98081. doi: 10.1371/journal.pone.0098081 (PMC4032235; doi:10.1371/journal.pone.0098081)
Supplement: Table S1 — Serum levels of biomarkers in high-proteinuria and low-proteinuria CKD control subgroups. (PDF) [file pone.0098081.s001.pdf]

Table S1. Serum levels of biomarkers in high-proteinuria and low-proteinuria CKD control subgroups

	**High-Proteinuria (N = 37)**	**Low-Proteinuria (N =42)**
IgA (mg/ml)	2.72 ± 0.83	2.69 ± 1.17
Gd-IgA1 (Units/ml)	162.6 ± 56.6	143.0 ± 58.7
Gd-IgA1-specific IgG (Units)	1.44 ± 0.10	1.47 ± 0.20
Gd-IgA1-specific IgA (Units)	0.411 ± 0.146	0.555 ± 0.612

Values are mean ± SD

High-Proteinuria subgroup, urinary protein/creatinine ratio ≥ 2.5g/g

Low-Proteinuria subgroup, urinary protein/creatinine ratio < 2.5g/g
